# Assessing Variability in Children’s Exposure to Contaminants in Food: A Longitudinal Non-Targeted Analysis Study in Miami, Florida

**DOI:** 10.3390/jox15010011

**Published:** 2025-01-14

**Authors:** Luciana Teresa Dias Cappelini, Olutobi Daniel Ogunbiyi, Vinícius Guimarães Ferreira, Mymuna Monem, Carolina Cuchimaque Lugo, Monica Beatriz Perez, Piero Gardinali, Florence George, Daniel M. Bagner, Natalia Quinete

**Affiliations:** 1 Institute of Environment, Florida International University, Miami, FL 33199, USA; ldiascap@fiu.edu (L.T.D.C.);; 2 Department of Chemistry and Biochemistry, Florida International University, Miami, FL 33199, USA; 3 Faculdade de Saúde Publica da USP, Departamento de Saúde Ambiental, São Paulo 01246-904, SP, Brazil; 4 Department of Mathematics & Statistics, Florida International University, Miami, FL 33199, USA; 5 Center for Children and Families, Florida International University, Miami, FL 33199, USA; dbagner@fiu.edu; 6 Department of Psychology, Florida International University, Miami, FL 33199, USA

**Keywords:** socio-economic variability, seasonal trends, anthropogenic contaminants, orbitrap mass spectrometry, chemometrics

## Abstract

Food is essential for human survival; however, food can be an important route of exposure to contaminants. This study investigated the presence and distribution of anthropogenic contaminants in food consumed by families with small children in South Florida, United States, evaluating seasonal and socio-economic variabilities in chemical composition. QuEChERS protocols, followed by non-targeted analysis (NTA) using an LC-Orbitrap HRMS system, were used for the comprehensive screening of organic contaminants. The compounds were annotated and identified with the Compound Discoverer (CD) software, and contaminant distributions were analyzed using boxplots and Principal Component Analysis (PCA). The results showed significant seasonal and socio-economic differences in contaminant distributions (*p* < 0.05). In the wet season, a predominance of polymers and surfactants, such as dodecanedioic acid and N-dodecylacrylamide, were found in food, which might be due to increased transport of industrial pollutants during increased precipitation, while plasticizers (e.g., bis(2-ethylhexyl) phthalate) and drugs (e.g., warfarin) were more prevalent during the dry season, which could be related to less dilution effects in this period. A higher abundance of 1-nitrosopiperidine, present in cured meats, was noted in food from upper socio-economic classes, while the lower class showed higher abundance of benzocaine, a common topical anesthetic.

## 1. Introduction

The global population is expected to reach 10.9 billion by 2100, a nearly 25% increase from current levels [1]. This rapid growth poses significant challenges, particularly in food production, as around 690 million people today already face hunger [2]. To meet the demand, agricultural practices have evolved, incorporating genetically modified crops, fertilizers, and pesticides to improve yield and resistance to pests [3]. However, the intensive use of chemicals in food production raises concerns about their potential health effects [3]. While food provides essential nutrients for growth, development, and the maintenance of vital bodily functions, it also serves as a major route for exposure to contaminants. Along the food supply chain, humans can be exposed to biological (e.g., bacteria, viruses, fungi), physical (e.g., metal fragments, bone, glass particles), and chemical (e.g., pesticides, toxic metals, radioactive substances, antibiotic residues) contaminants [4]. This impact is especially severe in children, who are in the developmental stage, making them more vulnerable to these contaminants due to their immature immune system and higher absorption of chemical substances, which can more significantly affect their physical and neurological development [5]. These contaminants can enter food at various stages, including production, processing, and preparation, posing serious health risks such as endocrine disruption, cancer, neurodegeneration, and chronic diseases [6,7].

In recent decades, the study of anthropogenic contaminants in food has revealed a significant evolution in composition and impact. In the past, concerns were mainly related to conventional pollutants, such as toxic trace metals (e.g., lead, mercury, and cadmium), organochlorine pesticides, and industrial waste, introduced into the environment as by-products of industrialization and intensive agriculture [8]. Currently, emerging contaminants include residues of pharmaceutical products, microplastics, endocrine disruptors, and chemicals present in cosmetics and cleaning products. These contaminants are often introduced into the environment through diffuse sources, such as effluents from wastewater treatment plants and leaching from landfills [9,10]. The growing concern about these new contaminants is related to their impacts on human and environmental health, making it essential to develop effective methods for their detection.

The determination of anthropogenic contaminants can be performed using several techniques, depending on their chemical nature. Among the most widely used, liquid chromatography (LC) coupled with different types of available detectors stands out, especially mass spectrometry [11]. Traditionally, regulated chemical contaminants are usually analyzed using targeted analysis. However, in recent years, monitoring a wide range of organic compounds has become necessary; in this context, non-targeted analysis (NTA) is gaining prominence. NTA is a comprehensive analytical approach that identifies known and unknown organic compounds in complex matrices employing high-resolution mass spectrometry (HRMS) [12,13,14,15,16]. This technique is particularly valuable for monitoring emerging pollutants and assessing their potential impact on ecosystems and human health [16].

Although NTA is frequently used to assess, discover, and monitor organic contaminants, one of its significant challenges is the confident identification of compounds’ structures due to the vast diversity of chemicals in environmental and food samples. For this purpose, several software and data processing tools are available such as Skyline [17], TracMass 2 [18], and Compound Discover (CD). CD is one of the most widely used commercial platforms, along with an Orbitrap MS system (Thermo Fisher Scientific, Waltham, MA, USA); it offers algorithms that automate and accelerate data processing, improving the accuracy of compound identification. Several studies have demonstrated the effectiveness of CD in various environmental and food analyses [19,20,21].

The aim of this research is to utilize liquid chromatography and high-resolution mass spectrometry, non-targeted analysis, Compound Discoverer software, and bioinformatics to identify and characterize the presence and distribution patterns (chemical variability) of organic contaminants in food samples within the greater Miami area (Miami-Dade County and Broward County), Florida, USA. By integrating these methodologies, this study seeks to establish a comprehensive framework for evaluating the environmental and public health impacts of these substances in a typical urban environment.

## 2. Material and Methods

### 2.1. Chemicals and Reagents

The solvents and reagents used in the sample preparation were of LC-MS grade, with comprehensive descriptions of all chemicals provided in the Supplementary Materials. Detailed information about the chemical properties of the quality control (QC) standards and isotopically labeled pharmaceutical standards (IS), including purity levels, log Kow values (indicating the octanol/water partition coefficient), monitored *m*/*z*, detection modes, and monoisotopic masses, is available in Table S1. The Supplementary Materials also include guidelines on the preparation and storage of the QC samples.

### 2.2. Collection and Storage Protocols for the Sampling Campaign

The research included 49 families with small children (13 months–8 years), focusing on evaluating their exposure to contaminants through dietary habits, resulting in the collection of 206 food samples in Miami-Dade and Broward County, South Florida, between May 2022 and February 2024. After applying the Python script to identify contaminants in the samples, we worked with data from 40 families and a total of 87 samples (Table S2). Written consent was obtained from the families prior to their participation, following approval by the institutional review board (IRB-21-0385). Samples were gathered for two consecutive weeks each month during both the wet and dry seasons, resulting in four collections per year, to assess the effects of seasonal rainfall and temperature variations in Florida on chemical composition. Food samples were stored in a freezer at −20 °C to preserve them and prevent degradation of the analytes. They were processed within a maximum of 30 days [22,23].

### 2.3. Food Sample Preparation

**Figure 1:**
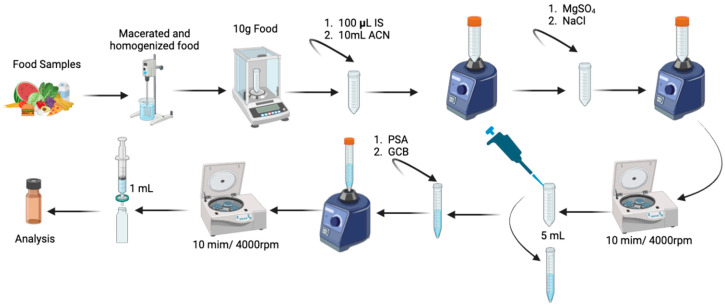
The extraction steps of food samples for subsequent analysis by LC-HRMS. Created with BioRender.com.

Frozen food samples were initially thawed and homogenized using a handheld immersion blender (KitchenAid Hand-held Blender, model KHBV53). Liquid samples, such as milk and infant formula, were mixed with solid food items (e.g., rice, carrots, beans, potato, broccoli, and chicken, among others) to facilitate homogenization before extraction. The Quick, Easy, Cheap, Effective, Rugged, and Safe (QuEChERS) method was employed to extract chemical compounds from the food matrix (Figure 1). Precisely, 10 g of each wet food sample (food items consumed by the children) was weighed and placed in a 50 mL Falcon tube, to which 100 µL of an internal standard (IS) solution was added, followed by 10 mL of acetonitrile, and was vortexed for 2 min. Subsequently, 1 g of NaCl and 4 g of MgSO_4_ were added to induce phase separation. The mixture was vortexed for 2 min and then centrifuged at 4000 rpm (2146 g) for 5 min. After centrifugation, 2 mL of the supernatant was transferred to a 15 mL Falcon tube, followed by the addition of 200 mg of Primary Secondary Amine (PSA) and 100 mg of Graphitized Carbon Black (GCB) for cleanup. This mixture was vortexed for 2 min and centrifuged again for 5 min at 4000 rpm (2146 g). Finally, 1 mL of the supernatant was filtered through an Acrodisc filter (0.2 μm, made of polyethersulfone) into a 2 mL LC vial for LC-HRMS analysis. This procedure was also applied to blanks and blanks spiked with QC samples [22,23].

### 2.4. Instrumental Analysis

Samples were analyzed by a liquid chromatography system (Vanquish, Thermo Scientific, USA) coupled to a Q-Exactive Orbitrap mass spectrometer (Thermo Scientific, USA) with a heated electrospray ionization (HESI) source, following the conditions outlined in previous studies [22,23]. Data were collected using full-scan and data-dependent acquisition (DDA) modes in both positive and negative ionization modes. Chromatographic separation was performed using a Hypersil GOLD aQ C18 column (100 × 2.1 mm, 1.9 µm, Thermo Scientific, USA). Twenty microliters of food extracts were directly injected into the liquid chromatography (LC) system for high-resolution mass spectrometry (HRMS) analysis. The LC method employed a mobile phase consisting of water (A), methanol (B), acetonitrile (C), and 0.1% formic acid (D). Blanks, quality controls (QCs), and samples were initially scanned from 100.0 to 800.0 m/z at a resolution setting of 140,000, followed by a data-dependent mode (dd-MS/MS) analysis at a resolution of 35,000 with a normalized collision energy of 30. Samples were injected four times and evaluated under both ionization conditions (positive and negative) using full scan and dd-MS2. Detailed specifics of the LC gradient program and mass spectrometry parameters are provided in Tables S3 and S4 [22,23].

### 2.5. Data Processing

The data processing workflow and associated criteria are described in the Supplementary Materials (Figure S1), utilizing an adapted CD workflow “Environmental Unknown ID w Online and Local Database Searches” within the software. This procedure includes peak detection and deconvolution, background noise reduction, feature consolidation, molecular formula determination, fragmentation analysis, isotopic pattern evaluation, elemental composition prediction, and database interrogation. The generated feature list was refined based on criteria including intensity thresholds, mass accuracy, signal-to-noise ratio, elemental composition, peak integrity, scoring, and pattern matching (Figure S2). Compound identification was restricted to level 2 on the Schymanski scale, ensuring that identifications were supported by substantial evidence such as library spectrum match, retention time (RT) alignment within a 2 min window of a predictive model, isotopic distribution, peak quality scores above 4, and sample peak areas at least three times greater than those of blanks. Features that did not meet these criteria were excluded. To reduce false positives, an in-house linear regression model predicting RT based on the log Kow was used to improve the accuracy of retention time predictions. Compound log Kow values were automatically retrieved from ChemSpider and PubChem databases via Python scripts, streamlining data collection and caching for efficiency [22,23].

### 2.6. Quality Assurance and Quality Control

Quality control (QC) samples containing chemicals with various polarities were analyzed in both positive and negative modes to ensure data integrity and optimal instrument performance. A QC solution with a concentration of 2 mg/L was prepared in methanol for both modes and stored at −20 °C (Table S1). Method blanks were processed using the same reagents, solvents and procedures as for sample extraction. QC checks were conducted after every two samples to evaluate instrument accuracy, with a minimum of 3–4 QCs per batch. The accuracy was greater than 70% for correctly identifying spiked QC standards. Performance graphs, previously published [23], were used to monitor and ensure the analytical method stayed within control limits. Any deviations beyond the defined limits prompted further testing and maintenance, such as replacing filters, preparing new solutions, and cleaning instrument components using specified procedures. Weekly mass calibrations with a dual-mode calibration solution ensured that the mass error remained below five ppm [22,23].

### 2.7. Statistical Analysis

The initial list consisted of several thousand compounds, including not only contaminants but also common organic compounds. To better address the purpose of this paper, an algorithm was written in Python to search PubChem for the chemical safety of each compound, in which only compounds classified as hazardous were considered for further analysis.

Statistical processing was performed in Python using libraries such as scipy.stats for statistical tests like Mann–Whitney U and Kruskal–Wallis, stats models.stats.multicomp for post hoc tests like Tukey, and scikit-learn for multivariate analysis. Initially, missing values were imputed with 1/5 of the lowest feature peak intensity. A logarithmic transformation was then applied to reduce the impact of large outliers, followed by feature-wise normalization by the sum. Finally, Pareto scaling was applied to ensure consistency in feature scales.

Univariate statistical analyses were conducted, with the choice of test based on the type of comparison. Mann–Whitney was used for binary comparisons, while Kruskal–Wallis was applied for comparisons involving multiple groups. Boxplots were plotted to visualize the differences. Finally, Principal Component Analysis (PCA) was performed for each comparison using statistically significant features (*p* ≤ 0.05) to assess sample grouping and discrepancies.

## 3. Results and Discussion

### 3.1. Chemical Seasonal Variability in Food

**Figure 2:**
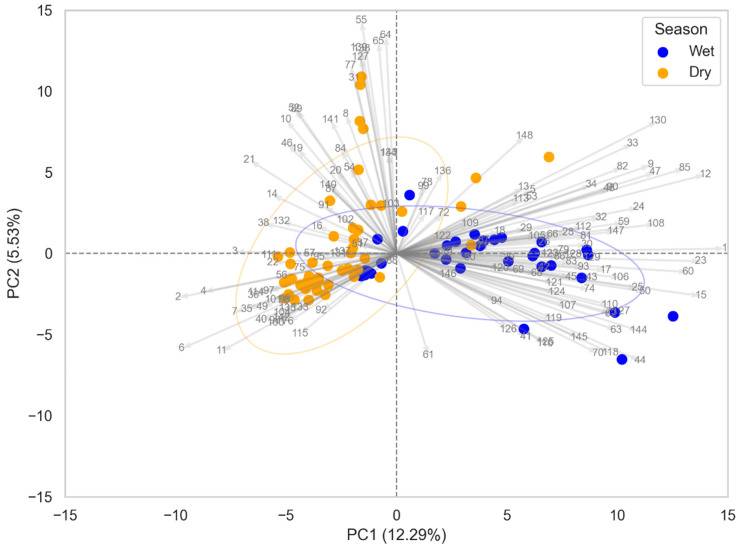
PCA biplot illustrating the separation of food samples collected in Miami, FL, based on the season. The blue dots represent samples collected during the wet season, while the yellow dots represent samples collected during the dry season. The arrows indicate the variable loadings, which contribute to the separation along the first two principal components (PC1: 12.29% and PC2: 5.53%). Ellipses represent the 95% confidence intervals for each seasonal group.

Food contamination is a public health issue that affects food safety on a global scale. Studies show that the presence of chemical contaminants can vary according to the season, significantly impacting food quality [24,25]. This phenomenon can be caused by various factors, such as climatic conditions that favor the resuspension of sediments and increase the leaching of pollutants from industrial and agricultural areas into water systems, among other factors [26]. In this study, we identified a variety of anthropogenic contaminants that played a crucial role in distinguishing food samples in Miami, FL, whereas 639 compounds were found in the dry (orange) and 367 compounds in the wet (blue) seasons (Figure S3), as illustrated in the PCA biplot (Figure 2).

The PCA was effective in scaling the data, highlighting the differences and similarities between the samples studied in two distinct categories. The principal components, PC1 and PC2, explained 12.29% and 5.53% of the variance, respectively, totaling 17.82% of the data variance. Although there was a good separation between the groups, some overlaps of samples were observed, suggesting that these samples may share similar chemical characteristics. The numbers shown in the PCA plot (Figure 2) correspond to the compounds used in this analysis, and their details are provided in Table S5 of the Supplementary Materials.

#### 3.1.1. Prevalent Chemicals in the Wet Season

**Figure 3:**
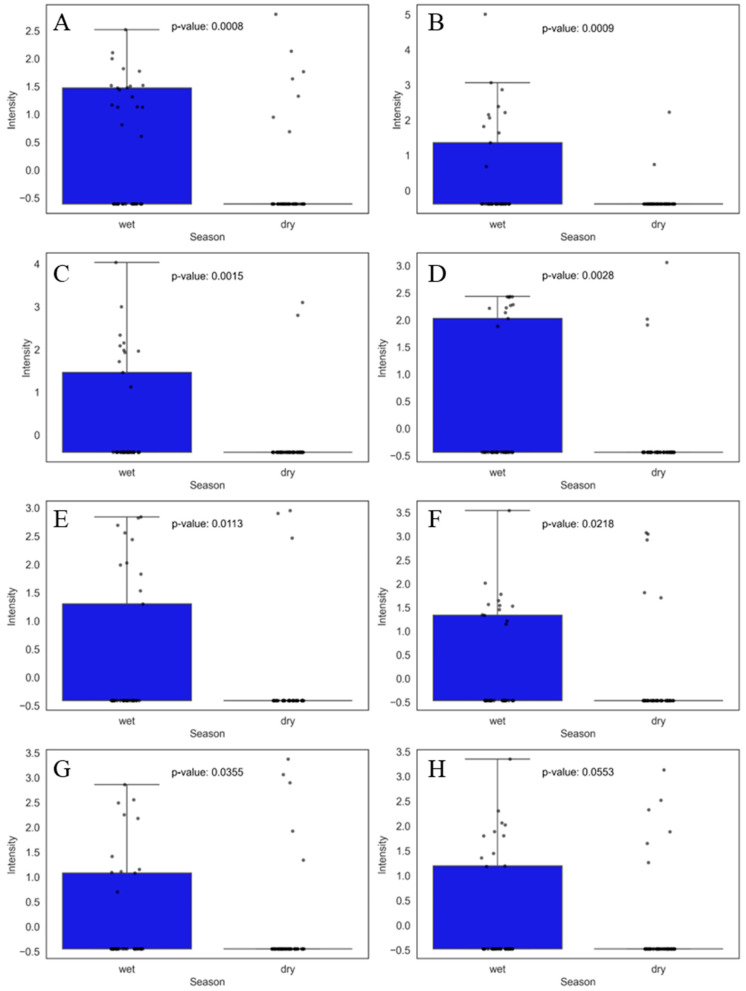
Boxplots of the eight main compounds that contributed the most to the separation of food samples during the wet season, as identified by PCA. Statistical significance was evaluated using the non-parametric Mann–Whitney U test, and the corresponding *p*-values are shown for each comparison. The boxplots display the distribution of compound levels and the differences between the evaluated groups. The upper whisker represents 1.5 times the interquartile range (IQR). Compounds are as follows: (**A**) N-dodecylacrylamide, (**B**) N-(aminopropyl)ethanolamine, (**C**) ethoxyquin, (**D**) dodecanedioic acid, (**E**) citral, (**F**) phenyl phosphate, (**G**) phenacetin, and (**H**) 2-oxindole.

Approximately eight compounds were present only in the wet season with a *p*-value ≤ 0.05 or only slightly above 0.05, as seen in Table S6 of the Supplementary Material. In this discussion, we highlight those that were most relevant to the study and had a potential correlation with food contamination. Among the compounds found, N-dodecylacrylamide, N-(aminopropyl)ethanolamine, ethoxyquin, dodecanedioic acid, citral, phenyl phosphate, phenacetin, and 2-oxindole were the most prevalent in the wet season, as shown in Figure 3.

N-dodecylacrylamide (Figure 3A) is part of a class of compounds known as N-alkylacrylamides, which are used in the formation of polymeric surfactants. These surfactants are characterized by their amphiphilic nature, which contains hydrophilic (water-attracting) and hydrophobic (water-repelling) components. This makes them effective in reducing surface tension and stabilizing dispersions in various applications, such as paints, inks, and water-based adhesives [27]. The other abundant compound found in the wet season was N-(aminopropyl)ethanolamine (Figure 3B) which is used in various chemical applications, including as an intermediate in synthesizing other chemicals. For this reason, it has been used in various industries as an emulsifier, surfactant, and polymer additive, in formulations of cleaning products and cosmetics, and in the pharmaceutical industry as an intermediate in the production of drugs or other bioactive products [28].

Furthermore, ethoxyquin, shown in Figure 3C, is a synthetic antioxidant used as an additive in animal feed and food preservation to prevent oxidation and rancidity. This compound, developed in the 1950s, has been widely used in animal feed, especially in poultry and fish feed, to prevent fat degradation and improve nutrient stability [29,30]. Studies have reported that ethoxyquin had toxic effects on zebrafish embryos (EC50 = 3.70 mgL^−1^) and daphnids (LC50 = 5.70 mgL^−1^) when exposed to this compound, and other studies have reported the presence of this compound and some of its metabolites in the tissues of pigs that consumed feed with this additive [29,30,31].

In addition to these, dodecanedioic acid (Figure 3D) is a compound with a high capacity to form bonds with other molecules. It has a wide range of applications, such as in the production of polymers and the lubricant industry, to improve thermal stability and resistance to oxidation. It can also be used in personal care products as an emollient or pH control agent, helping to soften and moisturize the skin [32,33,34]. Dodecanedioic acid has been studied as an alternative energy source, especially in metabolic conditions, such as for people with diabetes, due to its ability not to affect blood glucose levels [35]. There are no reports in the literature on the presence of this compound in food; however, some studies indicate that dodecanedioic acid was one of the metabolites found in rats after prolonged exposure to low doses of acrylamide, suggesting that it may be involved in metabolic disorders associated with chronic toxicity [35]. Another study mentioned that refined products of dodecanedioic acid must undergo rigorous purification steps to avoid the presence of toxic impurities [36].

The last four compounds with weight in the separation were citral (Figure 3E), phenyl phosphate (Figure 3F), phenacetin (Figure 3G), and 2-oxindole (Figure 3H). Citral has several applications, such as citrus fragrances in perfumes, cosmetics, and personal hygiene products; in the food industry, it is used as a flavoring in products such as beverages and sweets; and in the chemical industry, it can be used as an intermediate in the synthesis of ionone and vitamin A [37]. Phenyl phosphate is considered an industrially applicable compound, being a crucial intermediate in the synthesis of complex phosphate compounds for fertilizer, pharmaceutical, food, and beverage industries, among others [38]. Phenacetin is a compound that was widely used in the past as an analgesic and antipyretic. Some countries have banned its use due to its systemic toxicity, especially in the liver and kidneys [39]. In addition, studies have found this compound persistent in wastewater [40]. Wu and colleagues reported that phenacetin may influence the biological process in wastewater treatment [41]. 2-Oxindole is a compound that plays a crucial role in the synthesis of natural products, drugs, and other bioactive substances. Its versatile chemical structure allows modifications that result in a wide range of biological activities. Recently, there has been a significant increase in interest in 2-oxindole due to its potential use in the synthesis of pharmaceuticals, standing out as an essential building block in drug discovery programs [42].

The presence of these compounds during the wet season may be related to the increased leaching of pollutants from industrial and agricultural areas during rainfall, as well as the resuspension of sediments in water systems that may lead to greater dissemination of these compounds during this period [42]. In addition, the decomposition of organic residues due to microbial activity during wetter conditions may contribute to the transformation and release of these compounds into the environment [43].

#### 3.1.2. Prevalent Chemicals in the Dry Season

**Figure 4:**
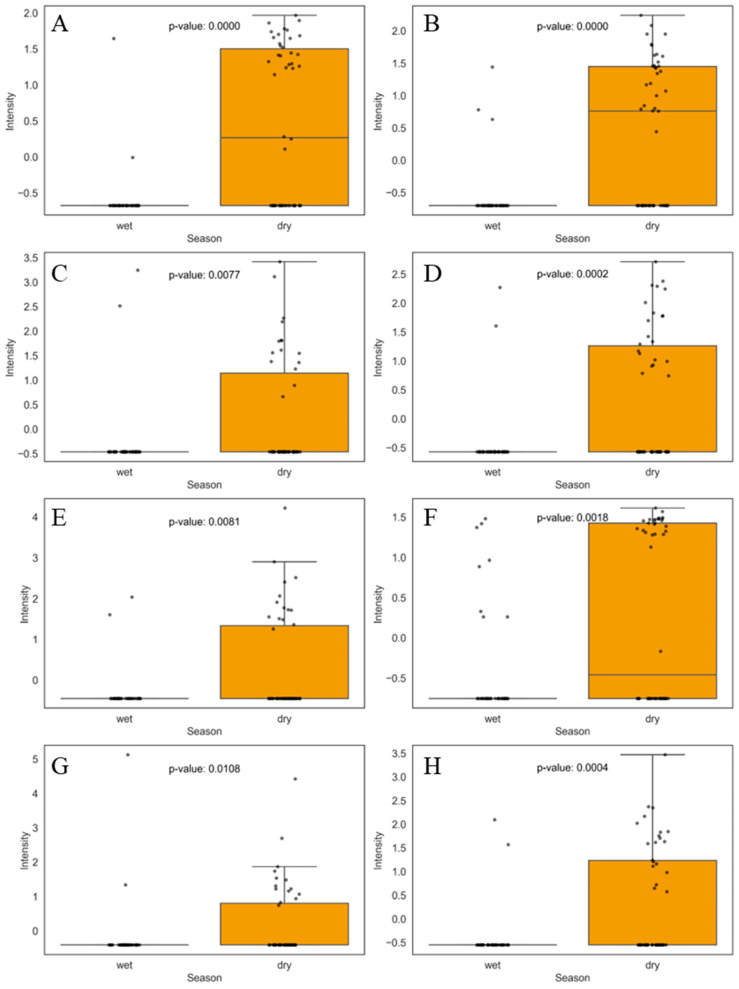
Boxplots of the eight main compounds that contributed the most to the separation of food samples during the dry season, as identified by PCA. Statistical significance was evaluated using the non-parametric Mann–Whitney U test, and the corresponding *p*-values are shown for each comparison. The boxplots display the distribution of compound levels and the differences between the evaluated groups. The upper whisker represents 1.5 times the interquartile range (IQR). Compounds are as follows: (**A**) bis(2-ethylhexyl) phthalate, (**B**) phthalic anhydride, (**C**) 2-tert-Butyl-9,10-anthraquinone, (**D**) warfarin, (**E**) etodolac, (**F**) 3,5-dibromopyridine-4-carbaldehyde, (**G**) imidazole-2-methanol, 1-methyl-, and (**H**) myristic acid.

Similarly to the wet season, approximately eight compounds were present only in the dry season with a *p*-value ≤ 0.05, as shown in Table S6 of the Supplementary Material. In this discussion, we highlight those more relevant to PCA separation according to the univariate analysis (boxplot) performed to identify these compounds. Among the most prevalent were bis(2-ethylhexyl) phthalate, phthalic anhydride, 2-tert-Butyl-9,10-anthraquinone, warfarin, etodolac, 3,5-dibromopyridine-4-carbaldehyde, imidazole-2-methanol, 1-methyl-, and myristic acid, as shown in Figure 4.

Bis(2-ethylhexyl) phthalate (DEHP) (Figure 4A) and phthalic anhydride (Figure 4B) were classified by univariate analysis as two of the main compounds responsible for the separation of food samples in the dry season. These compounds are used as plasticizers to manufacture polyvinyl chloride (PVC). DEHP is classified as an endocrine disruptor and has been associated with several adverse health effects, including reproductive and developmental toxicity, and is also related to oxidative stress and DNA damage [44]. Phthalic anhydride, in addition to being used to produce PVC, is also used in producing dyes, alkyd resins, and insecticides [45]. In addition, we have 2-tert-Butyl-9,10-anthraquinone (Figure 4C) used as a photoinitiator in the production of polymers and as an oxidizing agent in industrial processes [46]. These compounds can contaminate food via migration from plastic packaging materials [45,46,47].

In addition to polymers, the drugs warfarin (Figure 4D) and etodolac (Figure 4E) were also found in the food samples in the dry season. Warfarin is an anticoagulant used to reduce thromboembolic disorders [48], and etodolac is a nonsteroidal anti-inflammatory drug (NSAID) used to treat pain and inflammation [49]. Two other compounds, 3,5-dibromopyridine-4-carbaldehyde (Figure 4F) and imidazole-2-methanol, 1-methyl- (Figure 4G), are not drugs but are considered intermediates in the synthesis of other chemicals [50,51].

The concentration of pharmaceuticals in aquatic environments during the dry season tends to be higher due to a combination of environmental and behavioral factors. During dry periods, the volume of water in rivers, lakes, and reservoirs decreases significantly, which can concentrate pharmaceutical residues already present in the water. In addition, the reduction in water flow during the dry season limits the dilution of these compounds, leading to a higher relative concentration [52]. Furthermore, human behavior can influence the presence of these compounds, as certain medications may increase during dry or cold seasons due to the increase in related health conditions, such as chronic pain exacerbated by cold [53].

Finally, myristic acid (Figure 4H) is a saturated fatty acid in vegetable oils and animal fats and is particularly abundant in nutmeg oil [54]. This compound is also used in the cosmetics and personal care industry as an emollient, conditioning, and moisturizing agent [55]. Myristic acid can be found as a food additive, used to modify the texture and improve the stability of products [56]. Additionally, it is noticeable that in some cases, such as Figure 4A, 4B, and 4F, the median is clearly visible in the boxplot, while it appears to be absent for other compounds. This occurs because the median represents the middle value in a sorted sequence of data points. In cases where most values are clustered near the lower bound, close to zero, the median is also close to zero. When the median aligns with the lower quartile (25th percentile), it may not be visually distinguishable in the boxplot. The whisker bars represent 1.5 times the interquartile range (IQR), and values above this threshold can be considered outliers. Notwithstanding, outliers are not deleted in this study, since they can show potentially interesting exposure profiles.

### 3.2. Chemical Socio-Economic Variability in Food

**Figure 5:**
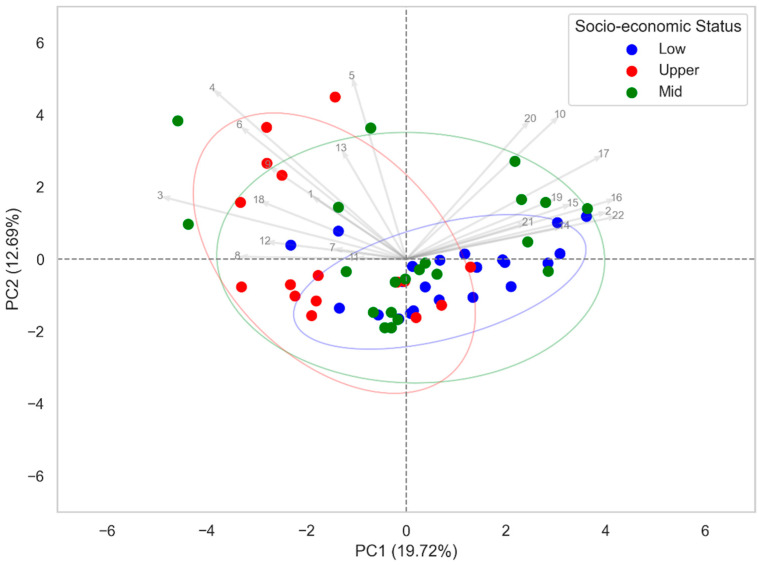
Biplot showing the separation of food samples collected in Miami, FL, based on the primary contaminants found. The blue dots represent samples from the low socio-economic group, red dots represent the upper socio-economic group, and green dots represent the middle socio-economic group. The arrows indicate the variable loadings contributing to the separation across the first two principal components (PC1: 19.72% and PC2: 12.69%). Ellipses represent the 95% confidence intervals for each socio-economic group.

Exposure to environmental contaminants varies significantly between different economic classes (Table S7). This can be due to geographic location, lifestyle, occupational activities, infrastructure, and more [57,58]. Studies have shown that economically disadvantaged populations are more exposed to contaminants such as fine particles and persistent chemicals due to their proximity to industrial areas, lower housing quality, and neighborhoods where they live. Individuals from upper socio-economic classes may be exposed to greater levels of pesticides and other organic contaminants due to their dietary habits and lifestyles, which often include consuming imported foods, fresh produce, and specialty and processed foods with a higher likelihood of contamination by such substances. The exposure of food to residues from cleaning products or materials used in house maintenance tends to be more significant within this class. These contaminants may ultimately reach food indirectly, either through cross-contamination during preparation or improper handling, or through the accumulation of these substances in the human body, followed by their excretion, which could contaminate the preparation environment and, consequently, the foods consumed [59,60]. In this study, we were also able to observe the presence of different anthropogenic contaminants in the food consumed by three different social classes in Miami, FL: 490 compounds in the low social class (blue), 253 compounds in the middle social class (green), and 115 compounds in the upper social class (red) (Figure S4), as illustrated in the PCA biplot (Figure 5). This analysis shows, overall, a good separation between the contaminants found in food consumed by children from low and upper socio-economic classes. However, the contaminants found in food from the middle social class were distributed among the other classes studied. These results can be observed in the PCA biplot (Figure 5), where the principal components, PC1 and PC2, explained 19.72% and 12.69% of the variance, respectively, totaling 32.41% of the data. The numbers shown in the PCA plot (Figure 5) correspond to the compounds used in this analysis, and their details are provided in Table S8 of the Supplementary Materials.

#### 3.2.1. Prevalent Chemicals in the Upper Socio-Economic Class

**Figure 6:**
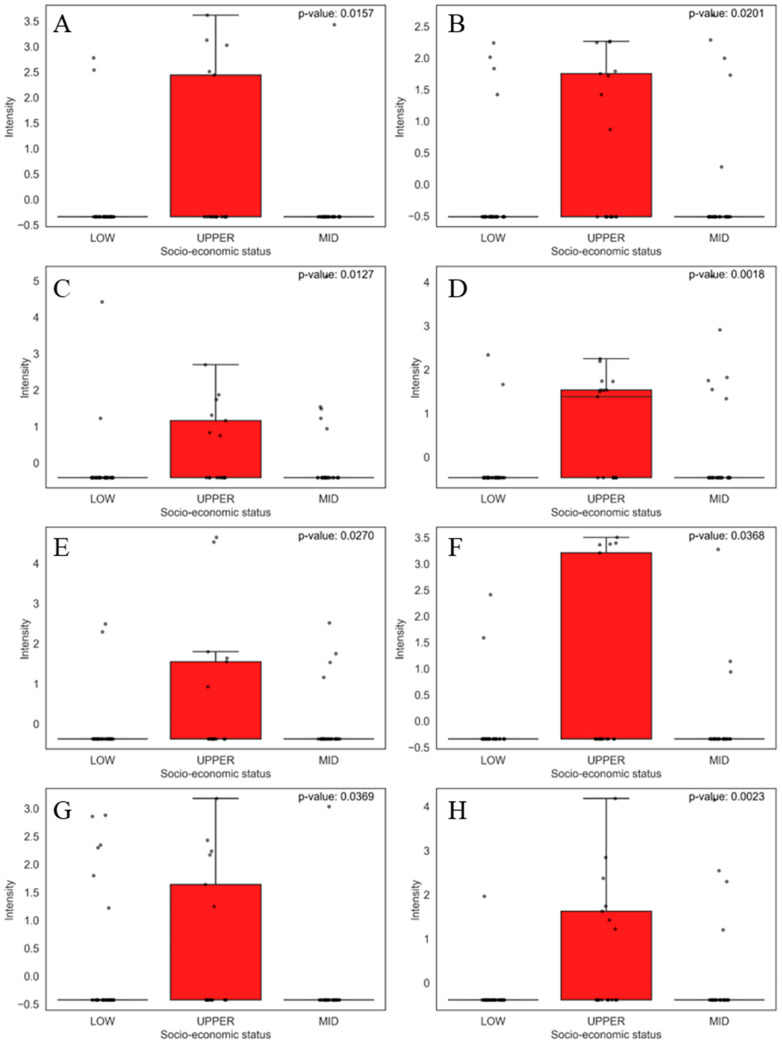
Boxplots of the eight main compounds that contributed the most to the separation of food samples in the upper socio-economic group, as identified by PCA. Statistical significance was evaluated using the non-parametric Mann–Whitney U test, and the corresponding *p*-values are shown for each comparison. The boxplots display the distribution of compound levels and the differences between the evaluated groups. The upper whisker represents 1.5 times the interquartile range (IQR). Compounds are as follows: (**A**) 1-nitrosopiperidine, (**B**) dichloroacetic acid, (**C**) imidazole-2-methanol, 1-methyl-, (**D**) 4,5-dicyano-2-aminoimidazole, (**E**) catechin, (**F**) hexylamine, (**G**) mellitic acid, and (**H**) 2-piperazinecarboxylic acid.

Among the abundant compounds found in the foods responsible for separating the samples from the upper socio-economic class were 1-nitrosopiperidine (Figure 6A), 1-methyl-imidazole-2-methanol (Figure 6B), and dichloroacetic acid (Figure 6C).

1-Nitrosopiperidine is a compound found in foods due to the nitrosation reaction when foods are preserved with nitrates and nitrites. These compounds are generally present in diets that include charcuterie and high-quality cured meats. Nitrosamines, such as 1-nitrosopiperidine, have potential carcinogenicity [61]. Another compound found was HMMNI (1-methyl-5-nitroimidazole-2-yl-methanol), which is associated with nitroimidazoles. It may be a food metabolite due to the use of antimicrobials, such as ronidazole (RNZ), in animals, which can bind to proteins in their bodies and thus enter the food chain [62]. Finally, we have dichloroacetic acid, which can be formed as a byproduct in foods during chlorination processes or because of the degradation of certain preservatives. An example would be bottled mineral water, which sometimes contains traces of dichloroacetic acid. The presence of this compound in foods may be a concern due to its carcinogenic effects [63].

The other relevant compounds in the separation of the univariate were 4,5-dicyano-2-aminoimidazole (Figure 6D), catechin (Figure 6E), hexylamine (Figure 6F), mellitic acid (Figure 6G), and 2-pyriperazinecarboxylic acid (Figure 6H).

There were no reports of 4,5-dicyano-2-aminoimidazole in the literature, but imidazoles are known to be formed during meat cooking [64]. Catechin is a flavonoid found in green tea and other plant foods. It is studied for its antioxidant properties and possible health benefits, such as cardiovascular protection [65]. Hexylamine is used to manufacture pharmaceuticals, dyes, and other industrial chemicals; it may be present in the environment due to industrial waste [66]. Toxicity studies in humans and animals have shown that it has neurotoxic and irritant effects observed in acute exposures [67]. Furthermore, mellitic acid and 2-piperazine carboxylic acid can be used in the synthesis of pharmaceutical products [68,69].

#### 3.2.2. Prevalent Chemicals in the Low Socio-Economic Class

**Figure 7:**
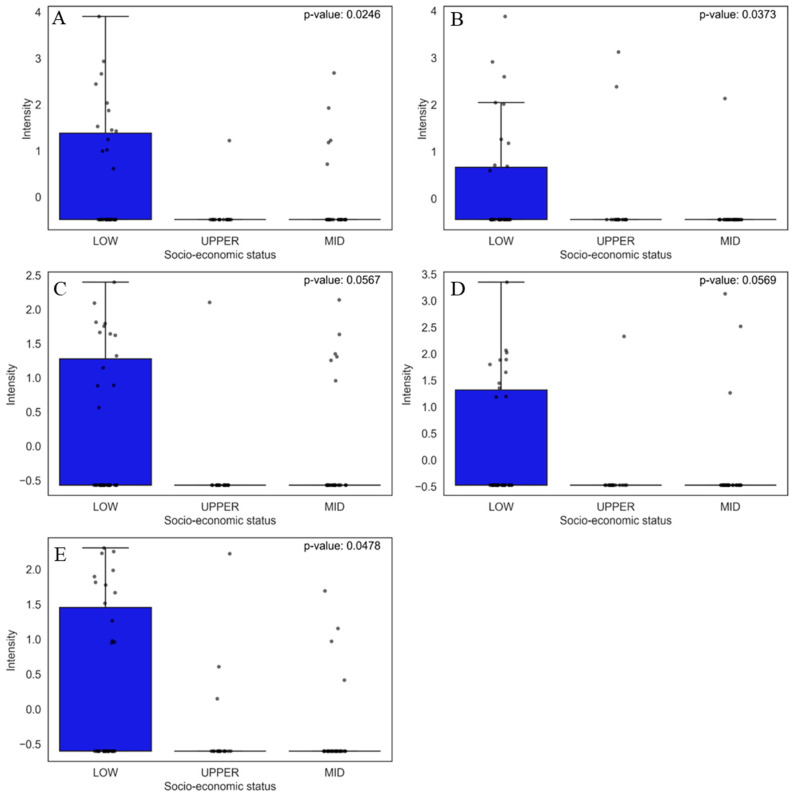
Boxplots of the five main compounds that contributed the most to the separation of food samples in the low socio-economic group, as identified by PCA. Statistical significance was evaluated using the non-parametric Mann–Whitney U test, and the corresponding *p*-values are shown for each comparison. The boxplots display the distribution of compound levels and the differences between the evaluated groups. The upper whisker represents 1.5 times the interquartile range (IQR). Compounds are as follows: (**A**) capsaicin, (**B**) 3-phenyllactic acid, (**C**) benzocaine, (**D**) 2-oxindole, and (**E**) tapentadol.

The most relevant compounds found in the univariate analysis to classify low socio-economic status were capsaicin (Figure 7A), 3-phenyl-lactic acid (Figure 7B), benzocaine (Figure 7C), 2-oxindole (Figure 7D), and tapentadol (Figure 7E).

Capsaicin is not considered a contaminant; it is an active compound present in peppers (Capsicum), widely consumed in several cultures, but its high consumption has been associated with health risks, such as an increased risk of gastric cancer [70]. The other compound found was 3-phenyl-lactic acid (3-PLA), commonly found in fermented foods such as pickles and cheeses, which can be produced naturally by lactic acid bacteria during the fermentation process [71] or be added to the process as a preservative [72].

In addition, benzocaine was found, which is not typically found in foods but is used as a topical anesthetic in over-the-counter products. However, contamination may be related to cross-contamination or food products produced in unregulated environments. There were no reports of the presence of benzocaine in food. However, studies by Meinertz [67] and collaborators showed that benzocaine is persistent in aquatic products [73], which may represent a risk of contamination in food from fish treated with this anesthetic. Another drug found was tapentadol, which is an analgesic that acts as an agonist of μ-opioid receptors and an inhibitor of norepinephrine reuptake. This drug is used to treat moderate to severe pain, including acute and chronic pain [74]. A study conducted by Barbosa et al. investigated the toxic effects of acute administration of tapentadol at effective analgesic doses and the maximum tolerated dose in rats, finding that this compound caused liver and kidney damage in the animals tested [75]. Meanwhile, 2-oxindole may be present in pharmaceutical and chemical products, including kinase inhibitors, anticancer drugs [76,77], and the synthesis of heterocyclic compounds generally used in various branches of industry, such as pigments, preservatives, and agrochemicals [77,78].

#### 3.2.3. Prevalent Chemicals in the Low and Upper Economic Classes

**Figure 8:**
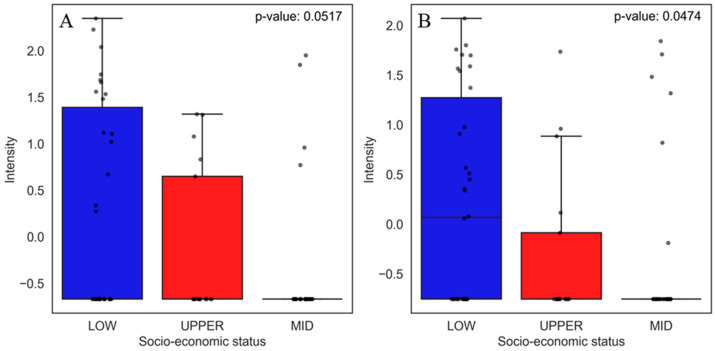
Boxplots of the two main compounds that contributed the most to the separation of food samples in the low and upper socio-economic groups, as identified by PCA. Statistical significance was evaluated using the non-parametric Mann–Whitney U test, and the corresponding *p*-values are shown for each comparison. The boxplots display the distribution of compound levels and the differences between the evaluated groups. The upper whisker represents 1.5 times the interquartile range (IQR). Compounds are as follows: (**A**) benalaxyl and (**B**) 2,2-methylenebis(4-ethyl-6-tert-butylphenol).

Although PCA (Figure 5) showed a good separation between the compounds found in food of the lower and upper socio-economic classes, two of them, benalaxyl (Figure 8A) and methylenebis(4-ethyl-6-tert-butylphenol) (MBEBP) (Figure 8B), were relevant in both social classes. The boxplots show that the low socio-economic class presents a more significant variability in the abundance (peak intensity) of these compounds compared to the upper socio-economic class, which shows more concentrated distributions and lower abundance.

Benalaxyl is a systemic fungicide of the acylanilide class widely used in agriculture to control fungal diseases in several crops, such as grapes, tomatoes, and potatoes. Its application has effectively managed pathogenic fungi, but recent studies raise concerns about its toxic effects and environmental behavior [79]. Research indicates that benalaxyl has stereoselective behavior, where its enantiomers present different degradation rates and toxicity. For example, the (-)-R-benalaxyl enantiomer is metabolized more rapidly than (+)-S-benalaxyl, resulting in more remarkable persistence of the more toxic enantiomer in the environment, which may increase ecotoxicological risks, especially in aquatic systems. Furthermore, prolonged exposure to this fungicide has been associated with oxidative stress in vital organs such as the liver and kidneys in animal models, highlighting the need for rigorous evaluation of its health and environmental impacts [80,81]. MBEBP is a synthetic antioxidant widely used in polymeric materials, including packaging that may come into contact with food. Studies in animal models indicate that MBEBP can cause a series of adverse effects, such as weight loss, anemia, and testicular atrophy in rats exposed to doses greater than 10 g/kg, in addition to acting as an uncoupler of oxidative phosphorylation in liver mitochondria, which may be related to its toxic effects [82,83].

#### 3.2.4. Prevalent Chemicals in the Middle Socio-Economic Class

**Figure 9:**
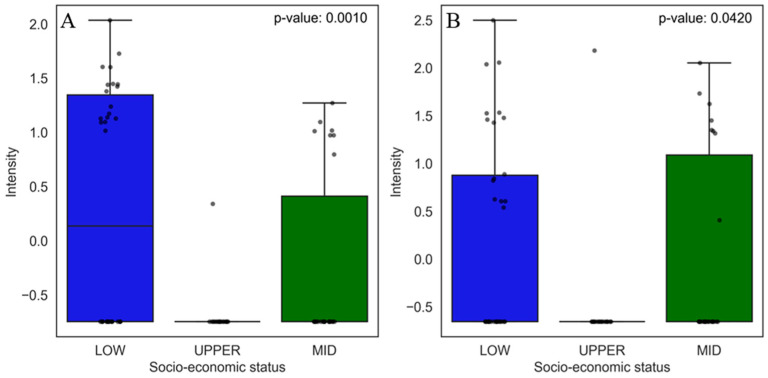
Boxplots of the two main compounds that contributed the most to the separation of food samples in the low and middle socio-economic groups, as identified by PCA. Statistical significance was evaluated using the non-parametric Mann–Whitney U test, and the corresponding *p*-values are shown for each comparison. The boxplots display the distribution of compound levels and the differences between the evaluated groups. The upper whisker represents 1.5 times the interquartile range (IQR). Compounds are as follows: (**A**) beta-ionone and (**B**) m-xylylenediamine.

As can be seen in the PCA (Figure 5), the compounds from the middle socio-economic class did not present good separation, having characteristics that kept them close to the x-axis of the PCA with the compounds from both the low and upper socio-economic classes. The main compounds between the low and middle socio-economic classes were beta-ionone (Figure 9A) and m-xylylene diamine (m-XDA) (Figure 9B). The boxplot shows that beta-ionone presents a higher abundance in the low socio-economic group, while the middle socio-economic group also shows some variability, but to a lesser extent. For m-xylenediamine, both groups present a slight variability between each other.

Beta-ionone is a compound found in several edible plants and used as a fragrance and flavor additive in food products. A study investigated beta-ionone’s toxicity and antimutagenic effects, highlighting that, although it is used in food, it can have biological effects that vary depending on the concentration and context of exposure [84]. m-Xylylenediamine (m-XDA) is a relevant chemical compound used in several industrial applications, including the production of polyamides, which are often used in food packaging due to their mechanical and barrier properties [85]. A European study established a method to determine m-XDA levels in food and highlighted the importance of controlling its migration into packaging, but did not perform toxicity tests to quantify its maximum and minimum intake limits [86].

**Figure 10:**
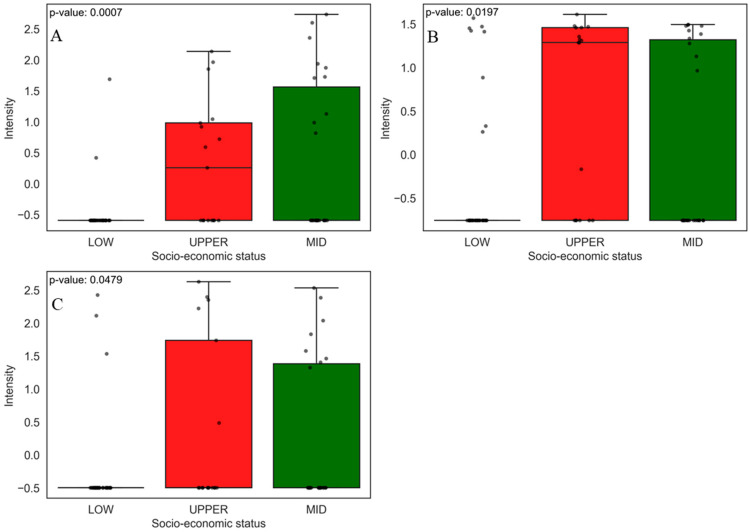
Boxplots of the three main compounds that contributed the most to the separation of food samples in the upper and middle socio-economic groups, as identified by PCA. Statistical significance was evaluated using the non-parametric Mann–Whitney U test, and the corresponding *p*-values are shown for each comparison. The boxplots display the distribution of compound levels and the differences between the evaluated groups. The upper whisker represents 1.5 times the interquartile range (IQR). Compounds are as follows: (**A**) NPYR, (**B**) 3,5-dibromopyridine-4-carbaldehyde, and (**C**) pellitorine.

Among the upper and middle socio-economic groups, the compounds 1-nitrosopyrrolidine (NPYR, Figure 10A), 3,5-dibromopyridine-4-carbaldehyde (Figure 10B), and pellitorine (Figure 10C) had a greater weight in the relationship between these classes. The boxplot shows that the NPYR compound is more abundant and dispersed in the middle socio-economic class. 3,5-Dibromopyridine-4-carbaldehyde has practically the same abundance between the two classes, and pellitorine is slightly more abundant in the upper socio-economic class.

NPYR is a nitrosamine frequently found in meats, processed dairy products, and alcoholic beverages [87]. A study by Jakszyn González [88] found a positive relationship between the intake of nitrosamine (such as NPYR) and the increased risk of gastric and esophageal cancer. The second compound found was 3,5-dibromopyridine-4-carbaldehyde, frequently used as an intermediate in synthesizing pharmaceuticals and agrochemicals [89]. Finally, we have pellitorine, a compound isolated from *anacyclus pyrethrum* that is known for its antimicrobial properties and use as a food flavoring agent. It can be present in spices and condiments and enhances flavor and aroma in processed products [90].

## 4. Conclusions

Our study demonstrates the importance of the comprehensive assessment of organic contaminants in food using advanced techniques such as non-targeted analysis (NTA) using LC coupled with high-resolution mass spectrometry (HRMS). It was possible to observe that seasonal variations can influence the chemical composition in food samples, highlighting the potential risks to public health and the environment. The PCAs revealed the significant presence of acrylamides, amino alcohols, synthetic antioxidants, dicarboxylic acids, monoterpenoid aldehydes, organophosphates, aromatic ethers, and heterocyclic compounds in the wet season, whereas in the dry season, phthalates, anhydrides, organic antioxidants, anticoagulants, anti-inflammatories, aldehydes, alcohols, and long-chain saturated fatty acids predominate. This fact can be explained by the physical and chemical properties of the compounds, as well as by the variations in emission sources and human activities associated with each season.

In addition, the research demonstrates that the distribution of emerging pollutants also differed across social classes, reflecting the interaction between socio-economic, geographic, and industrial factors. The upper and mid-upper classes are exposed to luxury products, high-end cosmetics, and fragrances, such as cyclic monoterpenoids (β-ionone) and substituted aromatic amines (xylylenediamine). The middle and low classes (mid–low) have a mix of exposure to medium-cost chemicals and pollutants, reflecting a balance between conscious consumption and affordability. Compounds that were found, such as alpha-hydroxycarboxylic acids (3-phenyllactic acid) and halogenated heterocyclic aldehydes (3,5-dibromopyridine-4-carbaldehyde), are common in skincare products and chemical synthesis intermediates used in affordable pharmaceuticals. The lower classes (low) are often exposed to higher concentrations of industrial pollutants and low-quality products such as diester phthalates (bis(2-ethylhexy) phthalate) and organophosphates (phenyl phosphate) that are used in low-cost plastics and affordable cleaning products, increasing exposure to potentially toxic substances. Prolonged exposure to these compounds can lead to significant oxidative stress, inflammation, neurotoxicity, reproductive harm, and increased risk of chronic diseases, including cancer and cardiovascular diseases.

The findings from this study provide a solid basis for future research and the formulation of public policies, highlighting the need for developing strategies to monitor and mitigate chemical pollution to effectively protect public health and the environment.

## Data Availability

The original contributions presented in this study are included in the article/supplementary material and the raw data supporting the conclusions of this article will be made available by the authors on request.

## Supplementary Materials

The following supporting information can be downloaded at: https://www.mdpi.com/article/10.3390/jox15010011/s1, Section S2.1: Chemicals and Reagents; Figure S1: Data processing steps workflow including detailed information on each node used in Compound Discoverer for identification of tentatively detected compounds; Figure S2: Non-targeted data processing approach to increasing confidence of tentatively detected features and application of Level 2 Schymanski scale; Figure S3: The Venn diagram illustrates the number of unique contaminants identified in each season (dry and wet), as well as the number of contaminants common to both seasons; Figure S4: The Venn diagram shows the number of unique contaminants identified in each socioeconomic class (low, middle, and upper), as well as the number of contaminants common to the different socioeconomic classes studied; Table S1: List of chemicals contained in the isotopically labeled standard (IS) and quality control (QC) mixtures, their molecular formula, log Kow, monitored ions, and monoisotopic mass; Table S2: Information on participating families and sample collection by season; Table S3: LC Gradient for direct injection to LC-HRMS (sample preparation offline); Table S4: MS scan parameters for NTA in positive and negative mode; Table S5: List of compounds corresponding to the numbers shown in the manuscript's PCA plot of Figure 2, for the wet and dry seasons; Table S6: Compounds identified in wet and dry seasons based on *p*-values from statistical analyses highlight significant differences in their distribution among the seasons; Table S7: Compounds identified in upper, middle, and lower social classes based on *p*-value from statistical analyses, highlighting significant differences in their distribution among distinct social classes; Table S8: List of compounds corresponding to the numbers shown in the manuscript’s PCA plot of Figure 5, categorized by socio-economic status.
