# Supplementary material for: Assessing Variability in Children’s Exposure to Contaminants in Food: A Longitudinal Non-Targeted Analysis Study in Miami, Florida
Source: J Xenobiot. 2025 Jan 14;15(1):11. doi: 10.3390/jox15010011 (PMC11755558; doi:10.3390/jox15010011)
Supplement: Supplementary file 1 [file jox-15-00011-s001.zip › jox-3299148-supplementary.pdf]

Assessing Variability in Children's Exposure to Contaminants in Food: A longitudinal Non-Targeted Analysis study in Miami, FL.

Luciana Teresa Dias Cappelini[a], Olutobi Daniel Ogunbiyi[a,b] , Vinícius Guimarães Ferreira[c], Mymuna Monem[d], Carolina Cuchimaque Lugo[a,b], Monica Beatriz Perez[a,e], Piero Gardinali[a,b], Florence George[d], Daniel M. Bagner[e,f], Natalia Quinete[a,b]*

[a] *Institute of Environment, Florida International University, Miami, FL, USA*

[b] *Department of Chemistry and Biochemistry, Florida International University, North Miami, FL*

[c] *Faculdade de Saúde Publica da USP, Departamento de Saúde Ambiental- São Paulo – SP Brazil*

[d] *Department of Mathematics & Statistics, Florida International University, Miami, FL, USA*

[e] *Center for Children and Families, Florida International University, Miami, FL, USA*

[f] *Department of Physiology, Florida International University, Miami, FL, USA*

******Correspondent* **Author:** nsoaresq@fiu.edu

Supplementary Information

Section S2.1 Chemicals and Reagents

Table S1. List of chemicals contained in the isotopically labeled standard (IS) and quality control (QC) mixtures, their molecular formula, log Kow, monitored ions, and monoisotopic mass.

Table S2. Information on participating families and sample collection by season.

Table S3. LC Gradient for direct injection to LC-HRMS (sample preparation offline).

Table S4. MS scan parameters for NTA in positive and negative mode.

Table S5: List of compounds corresponding to the numbers shown in the manuscript's PCA plot of Figure 2, for the wet and dry seasons.

Table S6. Compounds identified in wet and dry seasons based on p-values from statistical analyses highlight significant differences in their distribution among the seasons.

Table S7. Compounds identified in upper, middle, and lower social classes based on p-value from statistical analyses, highlighting significant differences in their distribution among distinct social classes.

Table S8: List of compounds corresponding to the numbers shown in the manuscript's PCA plot of Figure 5, categorized by socio-economic status.

Figure S1. Data processing steps workflow including detailed information on each node used in Compound Discoverer for identification of tentatively detected compounds.

Figure S2. Non-targeted data processing approach to increasing confidence of tentatively detected features and application of Level 2 Schymanski scale.

Figure S3. The Venn diagram illustrates the number of unique contaminants identified in each season (dry and wet), as well as the number of contaminants common to both seasons.

Figure S4. The Venn diagram shows the number of unique contaminants identified in each socioeconomic class (low, middle, and upper), as well as the number of contaminants common to the different socioeconomic classes studied.

2.1 Chemicals and Reagents

Acetonitrile, methanol, water (all Optima LC-MS grade), Florisil (500 g), graphitized carbon black (GCB, Superclean ENVI-Carb SPE bulk packing, pkg 50g), Cytiva Whatman acro disc filter (GMF, 0.2 µm), and Thermo Scientific™ Pierce™ calibration standards (positive and negative ESI (electrospray ionization)) were purchased from Fisher Scientific (Hampton, NH, USA). Formic acid (Optima LC/MS, 99.0+%) was purchased from Fisher chemical. Primary Secondary Amine (PSA, 100g, serial/lot #6645563-01) was purchased from Agilent Technologies (Santa Clara, CA, USA). Anhydrous Magnesium sulfate (99.5%, 500 g) was purchased from Alfa Aesar

(Haverhill, MA, USA), Sodium chloride (ACS certified, 10kg) was purchased from Fisher Scientific. Roche-b-Glucuronidase/Arylsulfatase enzyme (Helix pomatia, 10 mL, PAT: 10127698001) was purchased from Sigma Aldrich. Information on the isotopically labelled pharmaceutical standards (IS) utilized in this research is provided in Table S1. Food samples were spiked with 100 µL of a freshly prepared 5 µg/L internal standard mix in methanol. Quality control (QC) working solutions were prepared at concentration of 2 µg/L wase used for direct LC-HRMS injection. All standards (IS and QC) prepared in methanol were stored in the freezer at –20^{0}C. The chemical properties of the QC standards such as purity, logKow (octanol/water partition coefficient), monitored ions, detection modes and monoisotopic masses were included in Table S1.

Table S1. List of chemicals contained in the isotopically labeled standard (IS) and quality control (QC) mixtures, their molecular formula, log Kow, monitored ions, and monoisotopic mass.

Internal Standards	Purity, Source	Detection Mode	Log Kow	Monoisotopic Mass	Monitored Ions
**Trimethoprim-d9**	Toronto Research Chemicals	Positive	0.73	299.1944	300.2017
**Atenolol-d7**	Toronto Research Chemicals	Positive	-0.03	273.2070	274.2143
**Glipizide-d11**	Toronto Research Chemicals	Negative	3.35	456.2474	455.2401
**Warfarin-d5**	Toronto Research Chemicals	Negative	2.23	313.1362	312.1290
**QC Standards**	**Purity, Source**	**Detection Mode**	**Log Kow**	**Monoisotopic Mass**	**Monitored Ions**
**Caffeine**	>98.5%, Sigma	Positive	0.16	194.0804	195.0877
**Lincomycin**	>90%, Sigma	Positive	1.56	406.2137	407.2210

**Sulfamethoxazole**	>99%, Sigma	Positive	1.33	253.0521	254.0594
**Trimethoprim**	>98%, Sigma	Positive	1.69	290.1379	291.1452
**Norcocaine**	>99%, Cerilliant	Positive	1.96	289.1314	290.1387
**Carbamazepine**	>99%, Sigma	Positive	2.25	236.0950	237.1022
**(+)-cis-diltiazem hydrochloride**	>99%, Sigma	Positive	4.04	450.1380	415.1686
**Atrazine**	>98%, Sigma	Positive	2.82	215.0938	216.1010
**Diphenhydramine hydrochloride**	>98%, Sigma	Positive	3.11	291.1390	256.1696
**Fluoxetine hydrochloride**	100%, Sigma	Positive	4.65	345.1107	310.1413
**Sertraline hydrochloride**	>99%, Sigma	Positive	5.29	341.0505	306.0811
**Clotrimazole**	>98%, Sigma	Positive	6.26	344.1080	345.1153
**Sucralose**	99%, AK Scientific	Negative	-1.00	396.0146	395.0073
**Hydrochlorothiazide**	98.4%, MP Biomedicals	Negative	-0.1	296.9645	295.9572
**Diclofenac sodium**	100.38%, MP Biomedicals	Negative	4.02	316.9986	294.0094
**Gemfibrozil**	>99%, Sigma	Negative	4.77	250.1569	249.1496
**Mefenamic acid**	>98%, Sigma	Negative	5.28	241.1103	240.1030

Table S2. Information on participating families and sample collection by season.

Household Statistics			
Low	Middle	Upper	N/A
15	12	9	4
Child Statistics			
Girl		15	
Boy		25	
Under 12 Months	13-24 Months	25-36 Months	37-70 Months
0	20	6	14
Wet Season Samples		37	
Dry Season Samples		50	

Table S3. LC Gradient for direct injection to LC-HRMS (sample preparation offline).

Analytical pump- Direct Injection

Time	A%	B%	C%	D%	μL/min
0.0	0	0	2	98	250
2.9	0	0	60	40	250
4.9	0	40	60	0	250
10	0	40	60	0	250
11	0	0	60	40	250
12.5	0	0	2	98	250

A:LC-MS grade water, B: Methanol, C: Acetonitrile and D:0.1% formic acid

Table S4. MS scan parameters for NTA in positive and negative mode.

**Scan type**	Full MS	Full MS
**Scan range**	100.0 to 800.0 m/z	100.0 to 800.0 m/z
**Fragmentation**	None	None
**Resolution**	140000	140000
**Polarity**	Positive	Negative
**Microscans**	1	1
**Lock masses**	off	off
**AGC target**	3E6	3E6
**Maximum injection time (ms)**	200	200
**Sheath gas flow rate**	30	30
**Aux gas flow rate**	2	2
**Sweep gas flow rate**	1	1
**Spray voltage (kV)**	3.20	4.00
**Capillary temp (^{0}C)**	275	275
**S-lens RF level**	50.0	50.0

Table S5. List of compounds corresponding to the numbers shown in the manuscript's PCA plot of Figure 2, for the wet and dry seasons.

Number	Compound	RT (min)	m/*z*	Formula	Reference Ion
1	Beta ionone	7.723	193.15887	$C_{13}H_{20}O$	$[M+H]^+$
2	Bis(2-ethylhexyl) phthalate	11.032	391.2842	$C_{24}H_{38}O_4$	$[M+H]^+$
3	5-Bromo-4-methyl-2-Thiazolamine	8.223	190.92912	$C_4H_5BrN_2S$	$[M-H]^-$
4	Phthalic anhydride	9.417	149.0235	$C_8H_4O_3$	$[M+H]^+$
5	Choline	1.448	104.107	$C_5H_{13}NO$	$[M+H]^+$
6	4,5-dicyano-2-aminoimidazole	1.824	114.0209	$C_5H_3N_5$	$[M-H-H_2O]^-$
7	Warfarin	5.173	309.116	$C_{19}H_{16}O_4$	$[M-H]^-$
8	Myristic acid	8.678	227.2022	$C_{14}H_{28}O_2$	$[M-H]^-$
9	Pyrogallol	5.912	127.03879	$C_6H_6O_3$	$[M+H]^+$
10	2-Bromo-5-methyl-1,3,4-oxadiazole	7.482	160.93626	$C_3H_3BrN_2O$	$[M-H]^-$
11	Tris(2-ethylhexyl) trimellitate	12.16	547.39911	$C_{33}H_{54}O_6$	$[M+H]^+$
12	N-Dodecylacrylamide	7.912	240.2322	$C_{15}H_{29}NO$	$[M+H+MeOH]^+$

13	N-(Aminopropyl)Ethanolamine	1.378	119.118	$C_5H_{14}N_2O$	$[M+H]^+$
14	3-Amino-4-Cyano-5-Cyanomethylpyrazole	1.857	146.04694	$C_6H_5N_5$	$[M-H]^-$
15	{4-[(Vinyloxy)methyl]cyclohexyl}methanol	6.922	171.1379	$C_{10}H_{18}O_2$	$[M+H]^+$
16	5K8XI641G3	1.848	111.09167	$C_6H_{10}N_2$	$[M+H]^+$
17	Ethoxyquin	1.221	104.1069	$C_5H_{13}NO$	$[M+H]^+$
18	N-Acetyl-tyramine	5.171	180.10197	$C_{10}H_{13}NO_2$	$[M+H]^+$
19	M-trifluoromethylhippuric acid	3.953	246.03854	$C_{10}H_8F_3NO_3$	$[M-H]^-$
20	1D-chiro-inositol	1.966	225.06221	$C_6H_{12}O_6$	$[M+FA-H]^-$
21	3,5-Dibromopyridine-4-carbaldehyde	3.141	261.8512	$C_6H_3Br_2NO$	$[M-H]^-$
22	Erucic amide	10.86	338.34106	$C_{22}H_{43}NO$	$[M+H]^+$
23	Dodecanedioic acid	5.635	229.1446	$C_{12}H_{22}O_4$	$[M-H]^-$
24	(R)-3-Hydroxy myristic acid	7.706	243.19637	$C_{14}H_{28}O_3$	$[M-H]^-$
25	Methyl 3,5-di-tert-butyl-4-hydroxybenzoate	7.226	265.17969	$C_{16}H_{24}O3$	$[M-H]^-$
26	1-Benzosuberone	7.504	161.09608	$C_{11}H_{12}O$	$[M+H]^+$
27	Tetraiodoethylene	6.687	224.23712	$C_{15}H_{29}N$	$[M+H]^+$
28	Indole-3-ethanol	4.725	162.0912	$C_{10}H_{11}NO$	$[M+H+MeOH]^+$

29	Octyl methoxycinnamate	8.374	291.19508	$C_{18}H_{26}O_3$	$[M+H]^+$
30	6,7-Dihydroxy-4-methylcoumarin	5.096	193.04953	$C_{10}H_8O_4$	$[M+H]^+$
31	Carbamazepine	4.797	237.10188	$C_{15}H_{12}N_2O$	$[M+H]^+$
32	Bis(2-ethylhexyl)adipate	9.961	371.31453	$C_{22}H_{42}O_4$	$[M+H]^+$
33	Adenosine	2.268	268.10397	$C_{10}H_{13}N_5O_4$	$[M+H]^+$
34	Dopamine	1.612	154.0863	$C_8H_{11}NO_2$	$[M+H]^+$
35	1-Nitrosopiperidine	1.284	115.0868	$C_5H_{10}N_2O$	$[M+ACN+H]^+$
36	1-Amino-2-methyl-anthraquinone	2.041	119.54671	$C_{15}H_{11}NO_2$	$[M+2H]^{+2}$
37	3,6,9,12-Tetraoxahexacosan-1-ol	9.314	408.36792	$C_{22}H_{46}O_5$	$[M+NH4]^+$
38	2-tert-Butyl-9,10-anthraquinone	1.405	133.0650	$C_{18}H_{16}O_2$	$[M+H]^+$
39	Chrysin	3.852	206.04643	$C_{19}H_{19}ClO_8$	$[M+H]^+$
40	2-Piperazinecarboxylic acid	1.765	534.80426	$C_5H_{10}N_2O_2$	$[M+H]^+$
41	1-Boc-1,10-diaminodecane	6.06	273.25372	$C_{15}H_{32}N_2O_2$	$[M+H]^+$
42	N-Decanoylmorpholine	8.402	242.21127	$C_{14}H_{27}NO_2$	$[M+H]^+$
43	Veronal	1.737	226.11848	$C_8H_{12}N_2O_3$	$[M+H]^+$
44	Tert-butyl 3-(piperazin-1-yl) pyrrolidine-1-carboxylate	4.221	256.20178	$C_{13}H_{25}N_3O_2$	$[M+H]^+$

45	2-Propyl-1-indanone	7.455	157.10117	$C_{12}H_{14}O$	$[M+H-H_2O]^+$
46	Etodolac	5.728	288.1591	$C_{17}H_{21}NO_3$	$[M+H]^+$
47	Dodecyl acrylate	8.207	241.21651	$C_{15}H_{28}O_2$	$[M+H]^+$
48	4-Indolecarbaldehyde	5.467	144.04617	C_9H_7NO	$[M-H]^-$
49	Octocrylene	8.107	384.19406	$C_{24}H_{27}NO_2$	$[M+Na]^+$
50	2,5-di-tert-Butylhydroquinone	7.919	221.15489	$C_{14}H_{22}O_2$	$[M-H]^-$
51	Imidazole-2-methanol, 1-methyl-	8.767	329.2485	$C_5H_8N_2O$	$[M+H+MeOH]^+$
52	N,N-dimethylsulfamide	1.808	125.03771	$C_2H_8N_2O_2S$	$[M+H]^+$
53	Citral	7.559	153.1274	$C_{10}H_{16}O$	$[M+NH4]^+$
54	Nafronyl Oxalate	7.982	384.2529	$C_{24}H_{33}NO_3$	$[M+H]^+$
55	Hydralazine	3.975	159.0671	$C_8H_8N_4$	$[M-H]^-$
56	Etifoxine	8.082	299.0958	$C_{17}H_{17}ClN_2O$	$[M-H]^-$
57	4-Nitrosomorpholine	1.537	117.0658	$C_4H_8N_2O_2$	$[M+H]^+$
58	3-Phenoxybenzoic acid	5.985	213.0559	$C_{13}H_{10}O_3$	$[M-H]^-$
59	N-Caprylyldiethanolamine	5.03	218.2113	$C_{12}H_{27}NO_2$	$[M+H]^+$
60	Caprylic anhydride	11.357	283.2639	$C_{18}H_{36}O_2$	$[M+H]^+$
61	Trifloxystrobin	9.345	407.12109	$C_{20}H_{19}F_3N_2O_4$	$[M-H]^-$
62	(E,E)-2,4-Dodecadienal	7.209	181.1585	$C_{12}H_{20}O$	$[M+H]^+$

63	Dodecylsuccinic Anhydride	7.409	267.1964	$C_{16}H_{28}O_3$	$[M-H]^-$
64	2,5-Dimethyl-3-hexyne-2,5-diol	5.261	143.1068	$C_8H_{14}O_2$	$[M+H]^+$
65	Bis(trimethylsilyl)methane	4.508	159.1036	$C_7H_{20}Si_2$	$[M-H]^-$
66	3,5-Dihydroxybenzoic acid	3.707	153.0201	$C_7H_6O_4$	$[M-H]^-$
67	O-tert-Octylphenol	7.435	207.1741	$C_{14}H_{22}O$	$[M+NH_4]$
68	Dehydroabietic acid	7.805	301.2161	$C_{20}H_{28}O_2$	$[M+H]^+$
69	Valerophenone	6.559	163.1118	$C_{11}H_{14}O$	$[M+H]^+$
70	Dicyclohexylthiourea	7.224	241.17308	$C_{13}H_{24}N_2S$	$[M+H]^+$
71	Oxindole	4.555	134.06	C_8H_7NO	$[M+H]^+$
72	20alpha-Hydroxy-4-pregnen-3-one	8.227	315.2326	$C_{21}H_{32}O_2$	$[M+H]^+$
73	Tert-butyl 4-(cyanomethyl)piperidine-1-carboxylate	4.402	225.15944	$C_{12}H_{22}N_2O_2$	$[M+H]^+$
74	1-(Tetrahydro-2-furoyl)piperazine	3.903	185.1283	$C_9H_{16}N_2O_2$	$[M+H]^+$
75	Vanadium trioxide	1.816	148.86469	O_3V_2	$[M-H]^-$
76	Diethyl phosphate	1.697	153.03154	$C_4H_{11}O_4P$	$[M-H]^-$
77	N-Butyldiethanolamine	1.258	162.1486	$C_8H_{19}NO_2$	$[M+H]^+$
78	Dimethylphosphinic chloride	1.016	110.9767	C_2H_6ClOP	$[M-H]^-$
79	2,4-Quinolinediol	3.876	162.05492	$C_9H_7NO_2$	$[M+H]^+$
80	Fonofos	4.892	247.03658	$C_{10}H_{15}OPS_2$	$[M+H]^+$
81	Diethyl diallylmalonate	6.569	241.1429	$C_{13}H_{20}O_4$	$[M+H]^+$

82	1-Dodecyl-2-pyrrolidinone	8.402	254.2473	$C_{16}H_{31}NO$	$[M+H]^+$
83	Phenyl phosphate	1.179	327.0704	$C_{18}H_{15}O_4P$	$[M+H]^+$
84	NPYR	11.681	101.07088	$C_4H_8N_2O$	$[M+H]^+$
85	2,2-Methylenebis(4-ethyl-6-tert-butylphenol)	9.232	367.26367	$C_{25}H_{36}O_2$	$[M-H]^-$
86	Diphenylamine	6.79	170.0964	$C_{12}H_{11}N$	$[M+H]^+$
87	p-Decyloxyaniline	8.114	250.2162	$C_{16}H_{27}NO$	$[M+H]^+$
88	Methenolone	6.139	303.2313	$C_{20}H_{30}O_2$	$[M-H]^-$
89	Medronic Acid	0.151	174.9565	$CH_6O_6P_2$	$[M-H]^-$
90	O-Propanoylcarnitine	1.386	218.1384	$C_{10}H_{19}NO_4$	$[M-H]^-$
91	Guaiacolsulfonate	1.599	123.5248	$C_7H_8O_5S$	$[M-H]^-$
92	Hexylamine	8.448	371.10184	$C_6H_{15}N$	$[M+H]^+$
93	5-Methylcytosine	1.532	167.0927	$C_5H_7N_3O$	$[M+ACN+H]^+$
94	2,4-Dihydroxybenzoic acid	3.638	153.0201	$C_7H_6O_4$	$[M-H]^-$
95	Pentachloroethane	2.016	200.85962	C_2HCl_5	$[M+H]^+$
96	2-Chloro-6-fluorobenzoic acid	1.823	174.99492	$C_7H_4ClFO_2$	$[M+H]^+$
97	Bixin	7.497	198.11496	$C_{25}H_{30}O_4$	$[M+2H]^{+2}$
98	Fyrol DMMP	1.01	123.02144	$C_3H_9O_3P$	$[M-H]^-$
99	Fesoterodine	8.596	412.284	$C_{26}H_{37}NO_3$	$[M+H]^+$
100	MFCD00083078	6.075	354.3365	$C_{22}H_{45}NO_3$	$[M+H]^+$
101	Paxilline	8.596	412.284	$C_{26}H_{37}N_3$	$[M+2H]^{+2}$

102	Oxybutynin	7.672	358.237	$C_{22}H_{31}NO_3$	$[M+H]^+$
103	Norgestimate	8.084	370.23718	$C_{23}H_{31}NO_3$	$[M+H]^+$
104	Desoximetasone	8.682	377.21188	$C_{22}H_{29}FO_4$	$[M+H]^+$
105	Tyramine	1.75	138.09113	$C_8H_{11}NO$	$[M+H]^+$
106	Tert-butyl octahydropyrrolo[3,4-c]pyrrole-2-carboxylate	4.686	213.15973	$C_{11}H_{20}N_2O_2$	$[M+H]^+$
107	2,5-Furandicarboxylic acid	8.323	139.00252	$C_6H_4O_5$	$[M+H-H_2O]^+$
108	Phenacetin	4.935	180.1021	$C_{10}H_{13}NO_2$	$[M+H]^+$
109	Butoxytriglycol	4.414	207.1591	$C_{10}H_{22}O_4$	$[M+H]^+$
110	Lauryldimethylamine oxide	7.565	230.2475	$C_{14}H_{31}NO$	$[M+H]^+$
111	N,N-Bis(2-hydroxyethyl)dodecanamide	7.078	288.2533	$C_{16}H_{33}NO_3$	$[M+H]^+$
112	1-Naphthylmethylamine	4.314	158.09654	$C_{11}H_{11}N$	$[M+H]^+$
113	6-Benzylamino-1-hexanol	7.537	208.16945	$C_{13}H_{21}NO$	$[M+H]^+$
114	3-(Trifluoromethyl)-1H-1,2,4-triazol-5-amine	1.022	151.02359	$C_3H_3F_3N_4$	$[M-H]^-$
115	Pellitorine	5.141	102.06252	$C_{14}H_{25}NO$	$[M+H]^+$
116	L-Norleucine	1.345	132.1019	$C_6H_{13}NO_2$	$[M+H]^+$
117	5-Hydroxyindole-3-acetic acid	7.073	192.06551	$C_{10}H_9NO_3$	$[M+H]^+$
118	Pramiracetam	4.819	270.2175	$C_{14}H_{27}N_3O_2$	$[M+H]^+$
119	2,6-dimethylnaphthalene	7.291	157.1012	$C_{12}H_{12}$	$[M+H]^+$
120	Visnagin	5.964	231.0652	$C_{13}H_{10}O_4$	$[M+H]^+$

121	1-acetamidoadamantane	1.05	194.1535	$C_{12}H_{19}NO$	$[M+H]^+$
122	Cyclohexylurea	1.724	143.1179	$C_7H_{14}N_2O$	$[M+H]^+$
123	Boc-Ser(tBu)-OH	1.792	262.1647	$C_{12}H_{23}NO_5$	$[M+H]^+$
124	Tropinone	5.245	140.10703	$C_8H_{13}NO$	$[M+H]^+$
125	Gibberellic Acid	5.151	345.1354	$C_{19}H_{22}O_6$	$[M+H]^+$
126	Taurocholate	3.909	516.3013	$C_{26}H_{45}NO_7S$	$[M-H]^-$
127	N-Chloroacetyl-3-(trifluoromethyl)aniline	3.964	236.00996	$C_9H_7ClF_3NO$	$[M-H]^-$
128	1-acetylpiperidin-4-amine	1.06	143.11813	$C_7H_{14}N_2O$	$[M+H]^+$
129	Primidone	4.357	219.1129	$C_{12}H_{14}N_2O_2$	$[M+H]^+$
130	1-Methyl-2-azepanone	8.405	128.1068	$C_7H_{13}NO$	$[M+H]^+$
131	Allopurinol	1.551	137.0458	$C_5H_4N_4O$	$[M+H]^+$
132	Dichloroacetic acid	6.17	128.95085	$C_2H_2Cl_2O_2$	$[M+ACN+H]^+$
133	Diethyl phosphonomethanol	1.738	167.04733	$C_5H_{13}O_4P$	$[M-H]^-$
134	Heptanophenone	6.813	191.1431	$C_{13}H_{18}O$	$[M+H]^+$
135	2-(Trifluoroacetyl)thiophene	1.775	178.97858	$C_6H_3F_3OS$	$[M-H]^-$
136	bencyclane	8.279	290.24753	$C_{19}H_{31}NO$	$[M+H]^+$
137	5-Chlorothiophenesulphonyl chloride	12.312	214.88049	$C_4H_2Cl_2O_2S_2$	$[M-H]^-$
138	2-Aminonicotinic acid	1.112	139.0501	$C_6H_6N_2O_2$	$[M+H]^+$
139	3-Fluorovaline	9.268	413.2648	$C_5H_{10}FNO_2$	$[M-H]^-$
140	AZT	2.066	268.10452	$C_{10}H_{13}N_5O_4$	$[M+H]^+$

141	Triethyl orthoformate	7.285	233.2109	$C_{13}H_{28}O_3$	$[M+Na]^+$
142	Phosphinothricin	0.594	180.04259	$C_5H_{12}NO_4P$	$[M-H]^-$
143	Disulfide, bis(diisobutylthiocarbamoyl)	3.709	409.183	$C_{18}H_{36}N_2S_4$	$[M+H]^+$
144	4-(Aminoethyl)-1-N-Boc-piperidine	4.381	229.1909	$C_{12}H_{24}N_2O_2$	$[M+H]^+$
145	2,3-Diethyl-5-methylpyrazine	3.79	151.12297	$C_9H_{14}N_2$	$[M+H]^+$
146	Daidzin	5.713	417.1177	$C_{21}H_{20}O_9$	$[M+H]^+$
147	2-Oxindole	4.963	134.0601	C_8H_7NO	$[M+H]^+$
148	(2E,4E)-N-(2-methylpropyl)deca-2,4-dienamide	7.098	224.2004	$C_{14}H_{25}NO$	$[M+H]^+$
149	Dibutyl Phthalate	7.071	279.1589	$C_{16}H_{22}O_4$	$[M+H]^+$

RT: Retention Time; m/*z*: Mass-to-charge ratio.

Table S6. Compounds identified in wet and dry seasons based on p-values from statistical analyses highlight significant differences in their distribution among the seasons.

Compound	RT (mim)	m/*z*	Formula	Season
N-Dodecylacrylamide	7.912	240.2322	$C_{15}H_{29}$ NO	Wet
N-(Aminopropyl)Ethanolamine	1.378	119.118	$C_5H_{14}N_2O$	Wet
Ethoxyquin	1.221	104.1069	$C_5H_{13}NO$	Wet
Dodecanedioic acid	5.635	229.1446	$C_{12}H_{22}O_4$	Wet
Citral	7.559	153.1274	$C_{10}H_{16}O$	Wet
Phenyl phosphate	1.179	327.0704	$C_{18}H_{15}O_4P$	Wet
Phenacetin	4.935	180.1021	$C_{10}H_{13}NO_2$	Wet
2-Oxindole	4.963	134.0601	C_8H_7NO	Wet
Bis(2-ethylhexyl) phthalate	11.032	391.2842	$C_{24}H_{38}O_4$	Dry
Phthalic anhydride	9.417	149.0235	$C_8H_4O_3$	Dry
2-tert-Butyl-9,10-anthraquinone	1.405	133.0650	C_{18} $H_{16}O_2$	Dry
Warfarin	5.173	309.116	$C_{19}H_{16}O_4$	Dry
Etodolac	5.728	288.1591	$C_{17}H_{21}NO_3$	Dry

3,5-Dibromopyridine-4-carbaldehyde	3.141	261.8512	$C_6H_3Br_2NO$	Dry
Imidazole-2-methanol, 1-methyl-	8.767	329.2485	$C_5H_8N_2O$	Dry
Myristic acid	8.678	227.2022	$C_{14}H_{28}O_2$	Dry

RT: Retention Time; m/*z*: Mass-to-charge ratio.

Table S7: Compounds identified in upper, middle, and lower social classes based on p-value from statistical analyses, highlighting significant differences in their distribution among distinct social classes.

Compound	RT (mim)	m/*z*	Formula	Social - Class
NPYR	11.681	101.07088	$C_4H_8N_2O$	Upper-Mid
beta-lonone	7.723	193.15887	$C_{13}H_{20}O$	Low-Mid
4,5-dicyano-2-aminoimidazole	0.824	114.0209	$C_5H_3N_5$	Upper
2-Piperazinecarboxylic acid	0.857	131.08147	$C_5H_{10}N_2O_2$	Upper
Imidazole-2-methanol, 1-methyl-	8.767	329.2485	$C_5H_8N_2O$	Upper
1-Nitrosopiperidine	0.06	156.1134	$C_5H_{10}N_2O$	Upper
3,5-Dibromopyridine-4-carbaldehyde	3.141	261.8512	$C_6H_3Br_2NO$	Upper-Mid
Dichloroacetic acid	6.17	128.95085	$C_2H_2Cl_2O_2$	Upper
Capsaicin	6.321	306.2063	$C_{18}H_{27}NO_3$	Low
Catechin	3.872	289.07178	$C_{15}H_{14}O_6$	Upper
Hexylamine	8.448	371.10184	$C_6H_{15}N$	Upper
Mellitic acid	3.978	340.97918	$C_{12}H_6O_{12}$	Upper
3-Phenyllactic acid	4.254	165.05637	$C_9H_{10}O_3$	Low

m-Xylylenediamine	2.111	137.10736	$C_8H_{12}N_2$	Low/Mid
2,2-Methylenebis(4-ethyl-6-tert-butylphenol)	9.232	367.26367	$C_{25}H_{36}O_2$	Low-Upper
Tapentadol	7.323	222.18463	$C_{14}H_{23}NO$	Low
Pellitorin	5.141	102.06252	$C_{14}H_{25}NO$	Upper/Mid
Benalaxyl	14.024	326.17505	$C_{20}H_{23}NO_3$	Low-Upper
Benzocaine	3.46	166.08633	$C_9H_{11}NO_2$	Low
2-Oxindole	4.963	134.0601	C_8H_7NO	Low

RT: Retention Time; m/*z*: Mass-to-charge ratio.

Table S8. List of compounds corresponding to the numbers shown in the manuscript's PCA plot of Figure 5, categorized by socio-economic status.

Number	Compound	RT (min)	m/*z*	Formula	Reference Ion
1	NPYR	11.681	101.07088	$C_4H_8N_2O$	$[M+H]^+$
2	beta-lonone	7.723	193.15887	$C_{13}H_{20}O$	$[M+H]^+$
3	4,5-dicyano-2-aminoimidazole	1.824	114.0209	$C_5H_3N_5$	$[M-H-H2O]^-$
4	2-Piperazinecarboxylic acid	1.857	131.08147	$C_5H_{10}N_2O_2$	$[M+H]^+$
5	Imidazole-2-methanol, 1-methyl-	8.767	329.2485	$C_5H_8N_2O$	$[M+H+MeOH]^+$
6	1-Nitrosopiperidine	1.06	156.1134	$C_5H_{10}N_2O$	$[M+ACN+H]^+$
7	3,5-Dibromopyridine-4-carbaldehyde	3.141	261.8512	$C_6H_3Br_2NO$	$[M-H]^-$
8	Bromoacetic anhydride	8.621	377.2463	$C_4H_4Br_2O_3$	$[M-H]^-$
9	Dichloroacetic acid	6.17	128.95085	$C_2H_2Cl_2O_2$	$[M+ACN+H]^+$
10	Capsaicin	6.321	306.2063	$C_{18}H_{27}NO_3$	$[M+H]^+$
11	Catechin	3.872	289.07178	$C_{15}H_{14}O_6$	$[M-H]^-$
12	Hexylamine	8.448	371.10184	$C_6H_{15}N$	$[M+H]^+$
13	Mellitic acid	3.978	340.97918	$C_{12}H_6O_{12}$	$[M-H]^-$

14	3-Phenyllactic acid	4.254	165.05637	$C_9H_{10}O_3$	$[M-H]^-$
15	m-Xylylenediamine	2.111	137.10736	$C_8H_{12}N_2$	$[M+H]^+$
16	2,2-Methylenebis(4-ethyl-6-tert-butylphenol)	9.232	367.26367	$C_{25}H_{36}O_2$	$[M-H]^-$
17	Tapentadol	7.323	222.18463	$C_{14}H_{23}NO$	$[M+H]^+$
18	Pellitorin	5.141	102.06252	$C_{14}H_{25}NO$	$[M+H]^+$
19	{4-[(Vinyloxy)methyl]cyclohexyl}methanol	6.922	171.1379	$C_{10}H_{18}O_2$	$[M+H]^+$
20	Benalaxyl	14.024	326.17505	$C_{20}H_{23}NO_3$	$[M+H]^+$
21	Benzocaine	3.46	166.08633	$C_9H_{11}NO_2$	$[M+H]^+$
22	2-Oxindole	4.963	134.0601	C_8H_7NO	$[M+H]^+$

RT: Retention Time; m/*z*: Mass-to-charge ratio.

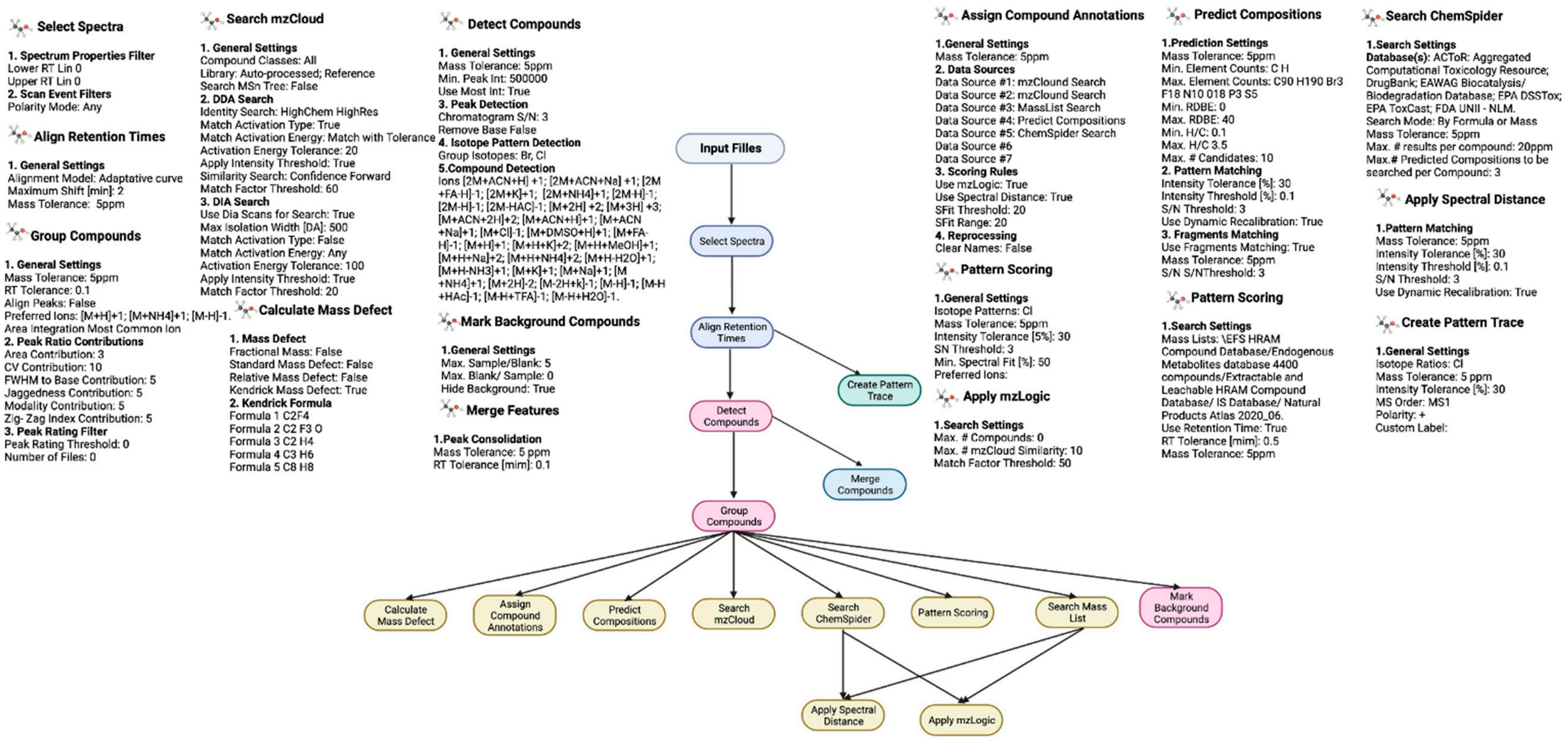

Figure S1. Data processing steps workflow including detailed information on each node used in Compound Discoverer for identification of tentatively detected compounds.

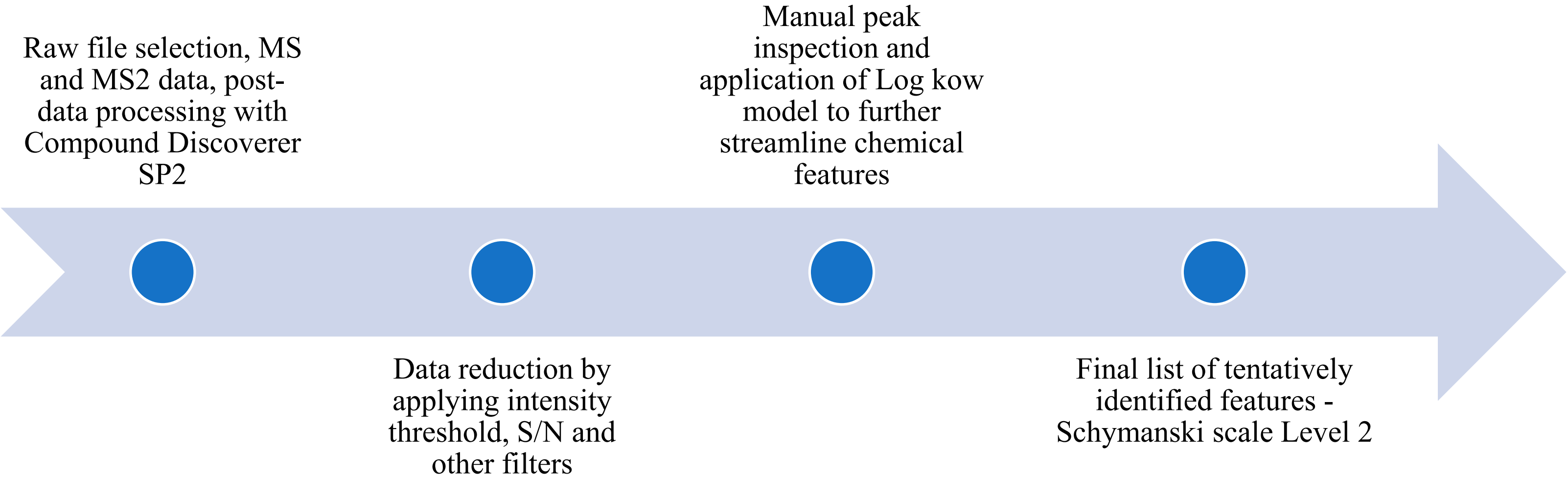

Figure S2. Non-targeted data processing approach to increasing confidence of tentatively detected features and application of Level 2 Schymanski scale.

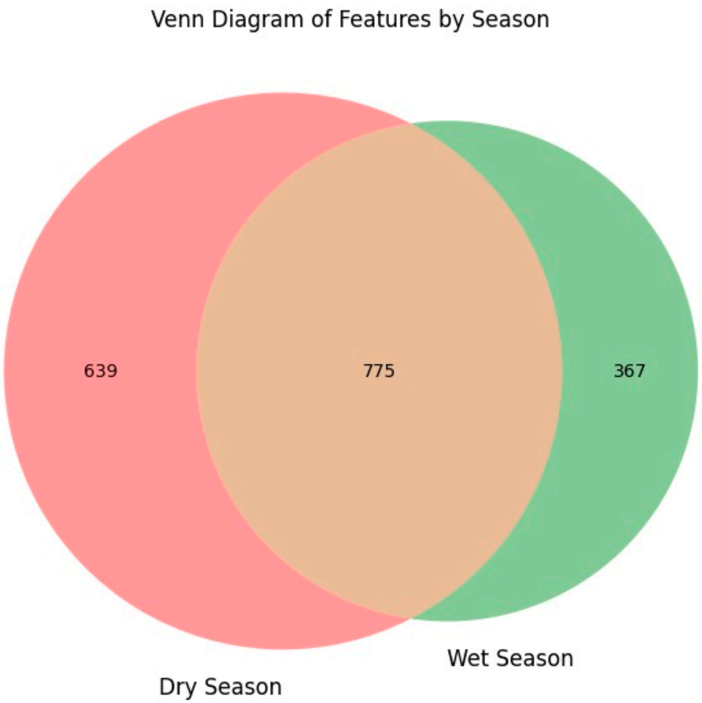

Figure S3. The Venn diagram illustrates the number of unique contaminants identified in each season (dry and wet), as well as the number of contaminants common to both seasons.

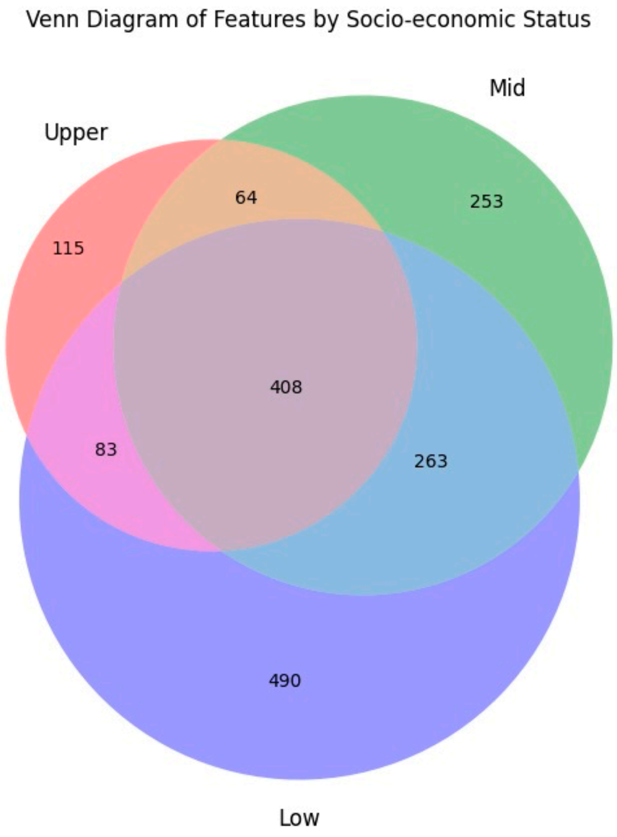

Figure S4. The Venn diagram shows the number of unique contaminants identified in each socioeconomic class (low, middle, and upper), as well as the number of contaminants common to the different socioeconomic classes studied.
